# Supplementary material for: Analysis of clinically relevant variants from ancestrally diverse Asian genomes
Source: Nat Commun. 2022 Nov 5;13:6694. doi: 10.1038/s41467-022-34116-9 (PMC9637116; doi:10.1038/s41467-022-34116-9)
Supplement: Supplementary file 1 — Supplementary Information [file 41467_2022_34116_MOESM1_ESM.pdf]

SUPPLEMENTARY INFORMATION

Analysis of clinically relevant variants from ancestrally diverse Asian genomes

Sock Hoai Chan, Yasmin Bylstra, Jing Xian Teo, Jyn Ling Kuan, Nicolas Bertin, Mar Gonzalez-Porta, Maxime Hebrard, Roberto Tirado-Magallanes, Joanna Hui Juan Tan, Justin Jeyakani, Zhihui Li, Jin Fang Chai, Yap Seng Chong, Sonia Davila, Liuh Ling Goh, Eng Sing Lee, Eleanor Wong, Tien Yin Wong, SG10K_Health Consortium, Shyam Prabhakar, Jianjun Liu, Ching-Yu Cheng, Birgit Eisenhaber, Neerja Karnani, Khai Pang Leong, Xueling Sim, Khung Keong Yeo, John C Chambers, E-Shyong Tai, Patrick Tan, Saumya S Jamuar, Joanne Ngeow, Weng Khong Lim

TABLE OF CONTENTS

SUPPLEMENTARY FIGURES 3

Supplementary Figure 1: Variant curation workflow. 3

Supplementary Figure 2: Pathogenic/Likely pathogenic variants in ACMG SF v3.0 list three genes associated with hereditary breast and ovarian cancers (*BRCA1, BRCA2, PALB2*) identified in the SG10K_Health cohort.. 4

Supplementary Figure 3: Carrier burden for prevalent autosomal dominant and recessive genes in Singaporean Chinese and Indian groups are correlated with gnomAD East Asian and South Asian populations respectively. 5

Supplementary Figure 4: Comparison of maxQ values between individuals of R/E-mismatched group (n=268) and R/E-matched group (n=8,783)... 6

Supplementary Figure 5: Strong concordance in gene-level carrier frequencies of samples sequenced to 15X and 30X target depth.. 7

SUPPLEMENTARY TABLES 8

Supplementary Table 1: Key details of the six participating studies aggregated under SG10K_Health project. 8

Supplementary Table 2: Demographic summary for 9,051 unrelated individuals included for analysis.. 9

Supplementary Table 3: Prevalence of P/LP variants in ACMG secondary findings (SF) genes by ancestry, sex and mode of inheritance... 10

Supplementary Table 4: Median values of genetic ancestral components of individuals with mismatched self-reported race/ethnicity (R/E) and ADMIXTURE-inferred genetic ancestry... 12

Supplementary Table 5: Association of the average fraction of ancestral component among individuals of Chinese, Indian and Malay ancestry groups with carriage status of pathogenic/likely pathogenic variants identified recurringly with the specified ancestral component. 13

Supplementary Table 6: SG10K_Health individuals at-risk of Centers for Disease Control and Prevention (CDC) Tier 1 genetic conditions and their respective pharmacogenetic profile for the drugs commonly prescribed for their condition. 14

SUPPLEMENTARY NOTE.. 15

SUPPLEMENTARY REFERENCES... 16

SUPPLEMENTARY FIGURES

Supplementary Figure 1: Variant curation workflow.

Variants that passed quality control metrics were curated according to the schema into four groups: pathogenic/likely pathogenic (P/LP) variants, variants of uncertain significance-favour pathogenic (VUS-FP), conflicting/uncertain variants (VUS), unclassified variants. pP: variants with probable pathogenic evidence, PTV: protein-truncating variants, sub: submitter.

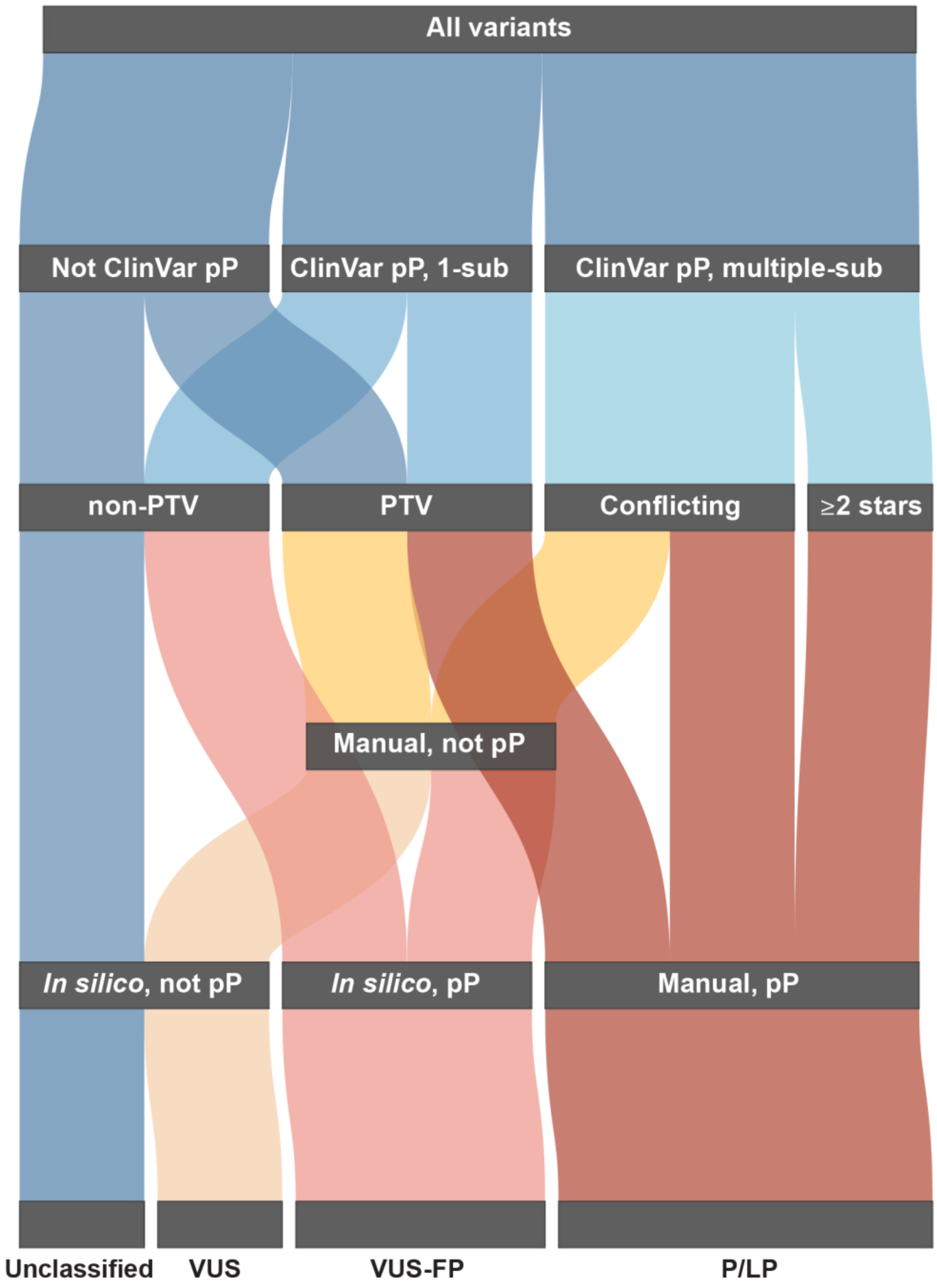

Supplementary Figure 2: Pathogenic/Likely pathogenic variants in ACMG SF v3.0 list three genes associated with hereditary breast and ovarian cancers (*BRCA1, BRCA2, PALB2*) identified in the SG10K_Health cohort.

Carrier frequency is adjusted to the size of each ancestry group (Chinese (CH): 5,502; Indian (IND): 1,941; Malay (MY): 1,608).

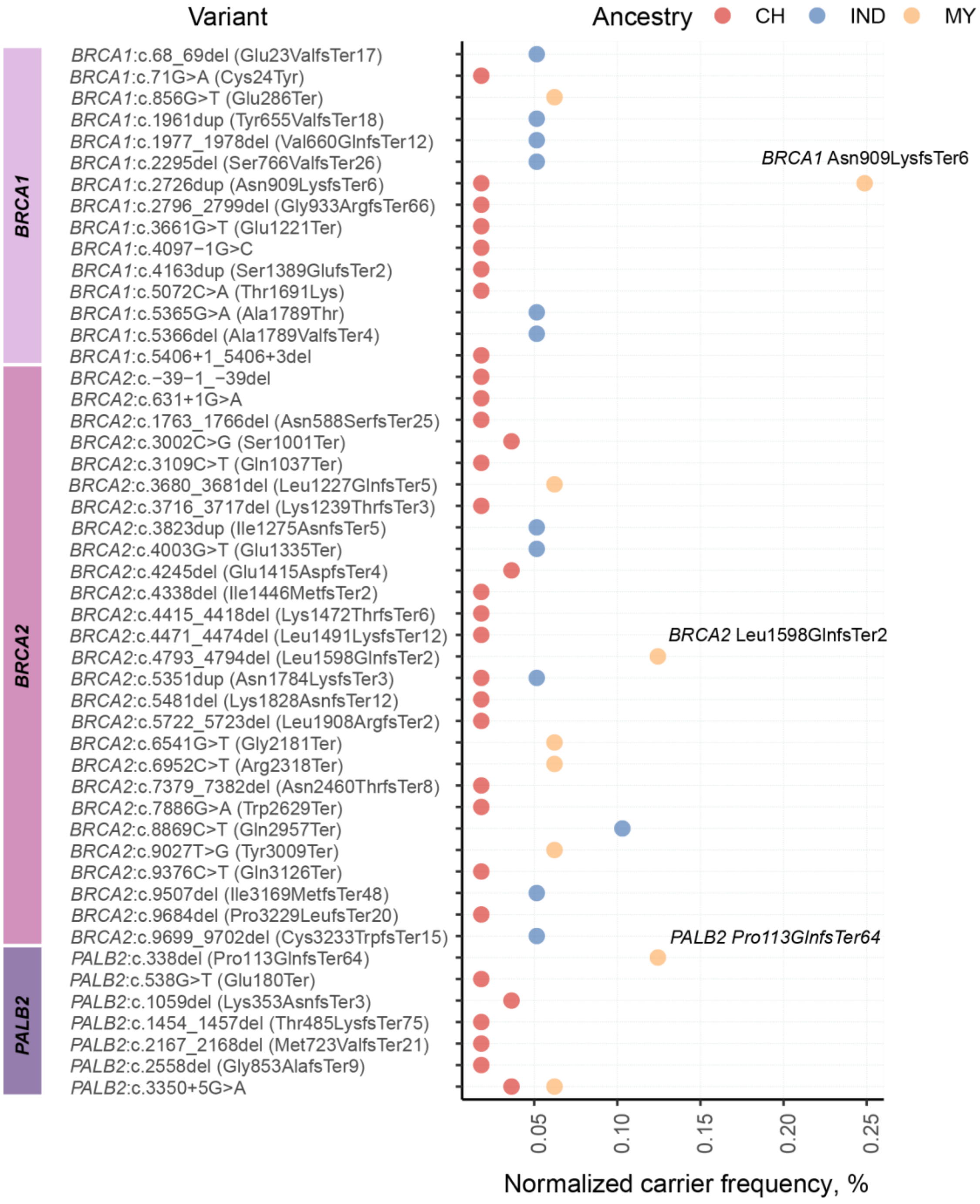

Supplementary Figure 3: Carrier burden for prevalent autosomal dominant and recessive genes in Singaporean Chinese and Indian groups are correlated with gnomAD East Asian and South Asian populations respectively.

Genes with carrier frequencies exceeding 0.5% in SG10K_Health Chinese (CH) or Indian (IND) groups were compared with gnomAD (v2.1) East Asian (EAS) or South Asian (SAS) groups, respectively. Correlation coefficient (*r*) and *p* value were calculated using Pearson's correlation method and the line was fitted using a linear regression model with 95% confidence interval displayed (shaded area).

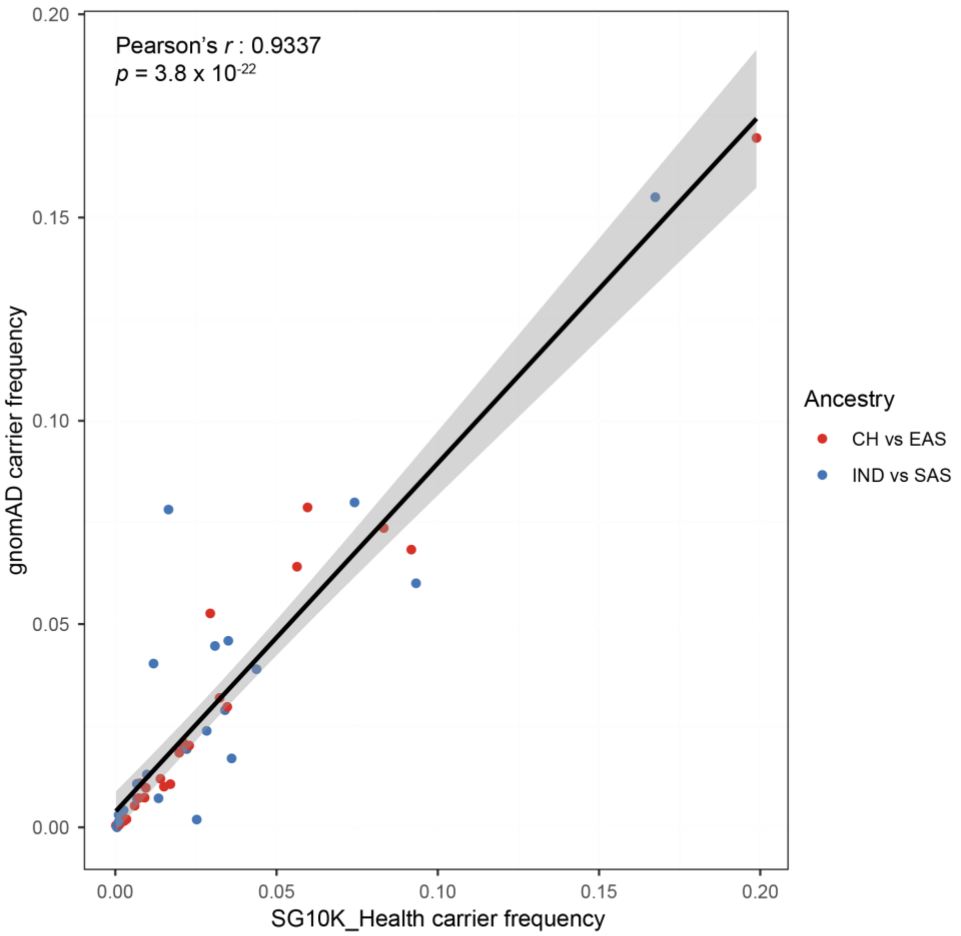

Supplementary Figure 4: Comparison of maxQ values between individuals of R/E-mismatched group (n=268) and R/E-matched group (n=8,783).

Individuals with mismatched self-reported and genetic ancestries are significantly more admixed as indicated by the lower median maxQ (0.53 vs 0.87), which is a measure of the highest ancestral component proportion. The box plot extends from the 25th to 75th percentiles and the length of the whiskers are defined as follows: upper whisker = min(maximum_value, Q3+1.5*IQR), lower whisker = max(minimum_value, Q1-1.5*IQR), where IQR is interquartile range, Q3 is third quartile, Q1 is first quartile. Horizontal line in the box represents the median. The overlapping violin plot shows the data distribution for each group. Statistical significance of difference between mismatched and matched groups was evaluated by two-sided Wilcoxon rank-sum test.

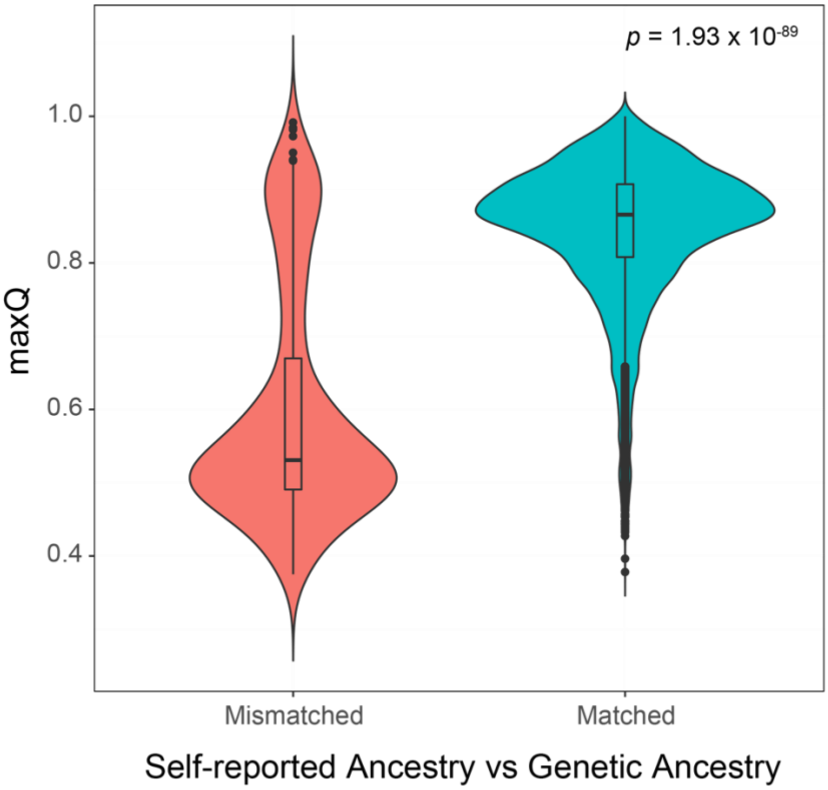

Supplementary Figure 5: Strong concordance in gene-level carrier frequencies of samples sequenced to 15X and 30X target depth.

Carrier frequencies for (a) autosomal dominant disorder genes and (b) recessive disorder genes were evaluated for 30X versus 15X samples of Chinese (CH, left panel), Indian (IND, middle panel) and Malay (MY, right panel) ancestry. Genes with carrier frequencies less than 10% were included for evaluation. (c) Carrier frequency of pharmacogenomic variants detected using ALDY (left panel), CYRIUS (middle panel) and VCF-derived (right panel) methods were strongly correlated for 30X versus 15X samples across all ancestry groups. Correlation coefficient (*r*) and *p* values were calculated using Pearson's correlation method and the line was fitted using a linear regression model with 95% confidence interval displayed (shaded area).

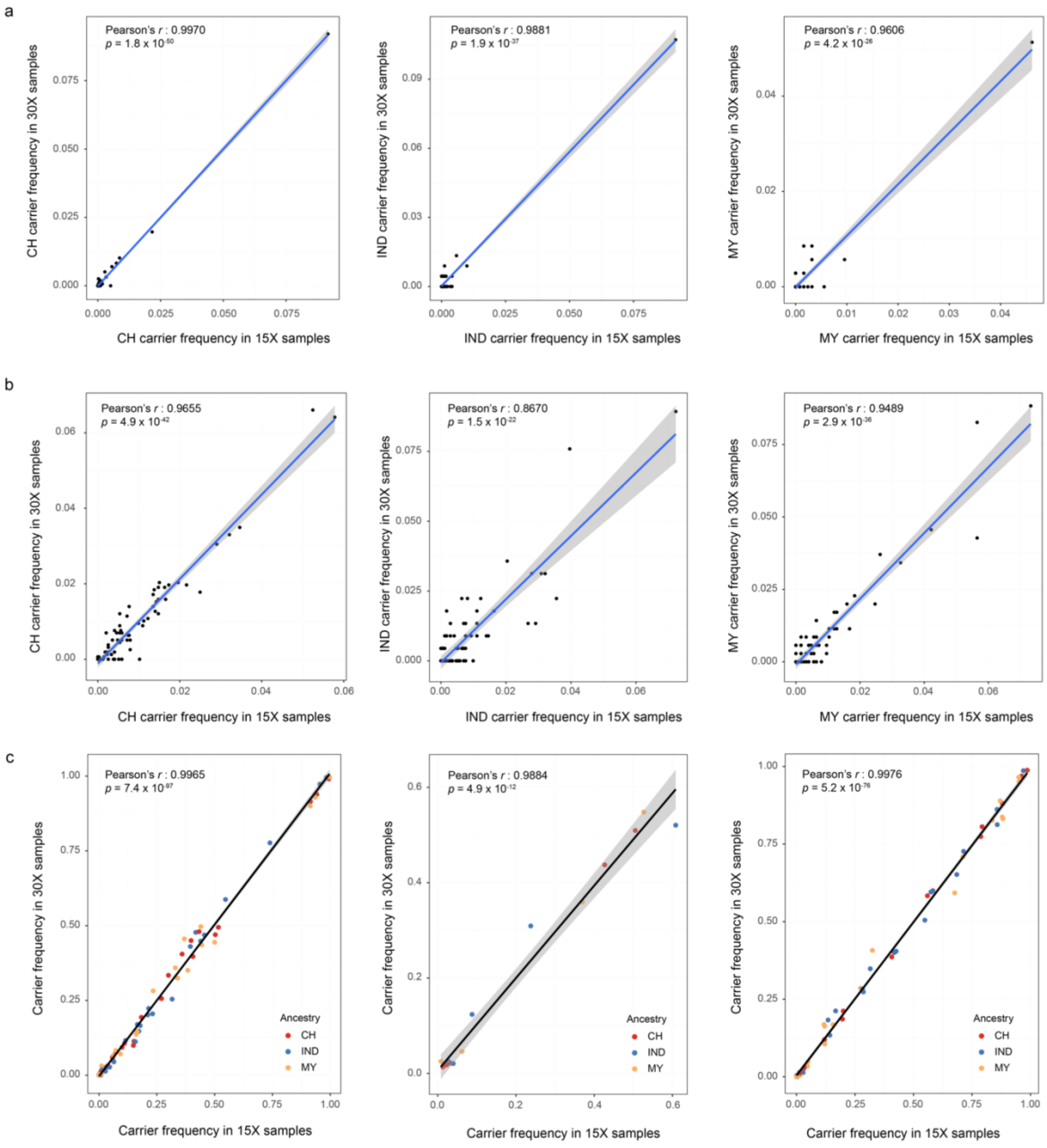

SUPPLEMENTARY TABLES

Supplementary Table 1: Key details of the six participating studies aggregated under SG10K_Health project.

Seq.: sequencing, NA: not applicable

Cohort Code	Study Name	Sample size [a]	Seq. coverage	Study IRB	Clinical trials identifier	Study reference
GUSTO	Growing Up in Singapore Towards healthy Outcomes birth cohort study	962 children	30x	National University Hospital, Singapore CIRB/E/2019/2655	NCT01174875	Soh et al. 2014 [1]
HELIOS	Health for Life In Singapore Study	1985 adults	15x	Nanyang Technology University, IRB 2016-11-030	NA	www.healthforlife.sg
MEC	Singapore Multi-Ethnic Cohort Study	2611 adults	15x	National University of Singapore, CIRB 13-512	NA	Tan et al. 2018 [2]
PRISM	SingHealth Duke-NUS Institute of Precision Medicine	1207 adults	30x	SingHealth Centralised Institutional Review Board , 2013/605/C	NCT02791152	Bylstra et al. 2019 [3]
SEED	Singapore Epidemiology of Eye Diseases study	1374 adults	15x	SingHealth Centralised Institutional Review Board, 2018/2717, 2018/2921, 2012/487/A, 2015/2279, 2018/2006, 2018/2594, 2018/2570	NA	Majithia et al. 2021 [4]
TTSH	Tan Tock Seng Hospital	912 adults	15x	National Health Group, TB-2020-001 & BTC-2020-001	NA	TTSH Personalized Medicine Normal Controls [b]

[a] The number of participants included in this study were not of the full cohort from each participating study but only those that were unrelated to the second degree.

[b] https://www.ttsh.com.sg/Patients-and-Visitors/Medical-Services/personalised-medicine/Pages/default.aspx

Supplementary Table 2: Demographic summary for 9,051 unrelated individuals included for analysis.

P/LP: pathogenic/likely pathogenic.

Characteristic	All		Chinese		Indian		Malay	
	n	(%)	n	(%)	n	(%)	n	(%)
No. individuals	9051		5502	60.8%	1941	21.4%	1608	17.8%
Sex								
Females	5182	57.3%	3161	57.5%	1106	57.0%	915	56.9%
Males	3869	42.7%	2341	42.5%	835	43.0%	693	43.1%
Age range (year)	birth - 85		birth - 85		birth - 83		birth - 84.8	
Age median [a] (year)	47		49		45		43	
No. individuals by age band								
birth-9 years	962	10.6%	566	10.3%	162	8.3%	234	14.6%
10-19 years	8	0.1%	6	0.1%	0	0	2	0.1%
20-29 years	549	6.1%	177	3.2%	174	9.0%	198	12.3%
30-39 years	1225	13.5%	708	12.9%	313	16.1%	204	12.7%
40-49 years	2544	28.1%	1422	25.8%	626	32.3%	496	30.8%
50-59 years	2226	24.6%	1455	26.4%	448	23.1%	323	20.1%
60-69 years	1197	13.2%	899	16.3%	178	9.2%	120	7.5%
70-79 years	273	3.0%	207	3.8%	38	2.0%	28	1.7%
80-89 years	19	0.2%	15	0.3%	1	0.1%	3	0.2%
Unknown	48	0.5%	47	0.9%	1	0.1%	0	0
No. carriers of P/LP variant [b]	7385	81.6%	4626	84.1%	1505	77.5%	1254	78.0%

[a] bimodal peak at birth and 47.2y

[b] P/LP variants identified in our evaluated gene-set of 4,143 genes

Supplementary Table 3: Prevalence of P/LP variants in ACMG secondary findings (SF) genes by ancestry, sex and mode of inheritance.

For AD conditions, prevalence in three key disease domains were tabulated. Pairwise comparison of carrier frequencies across ancestry groups were evaluated using two-sided Fisher's exact test, with *p* values adjusted using Benjamini-Hochberg correction. Highlighted in bold fonts are statistically significant $p < 0.05$. AD: autosomal dominant, AR: autosomal recessive, XL: X-linked, CH: Chinese, IND: Indian, MY: Malay, N: Total number of individuals in the group, n: number of P/LP carriers in the group, CVD: cardiovascular disorder, pred.: predisposition.

Feature		All (N = 9,051)		CH (N = 5,502)		IND (N = 1,941)		MY (n = 1,608)		Adjusted *p* value		
		n	(%)	n	(%)	n	(%)	n	(%)	CH : IND	CH : MY	IND : MY
ACMG SF v3.0 (73 genes)												
All genes		1392	(15.38)	693	(12.60)	531	(27.36)	168	(10.45)	6.6E-47	2.12E-02	7.57E-38
AD genes		309	(3.41)	191	(3.47)	65	(3.35)	53	(3.30)	1	1	1
	by no. variants:											
	• 1 P/LP	307	(3.39)	190	(3.45)	64	(3.30)	53	(3.30)	-	-	-
	• 2 P/LP	2	(0.02)	1	(0.02)	1	(0.05)	0		-	-	-
	by disease domain:											
	• Cancer pred.	97	(1.07)	59	(1.07)	18	(0.93)	20	(1.24)	6.96E-01	6.96E-01	6.96E-01
	• CVD	128	(1.41)	68	(1.24)	39	(2.01)	21	(1.31)	5.78E-02	8.00E-01	1.76E-01
	• Lipid disorder	65	(0.72)	58	(1.05)	3	(0.15)	4	(0.25)	**7.93E-05**	**1.70E-03**	7.09E-01
AR genes [a]		1124	(12.42)	512	(9.31)	490	(25.24)	122	(7.59)	**1.61E-62**	**3.25E-02**	**3.03E-46**
XL genes		15	(0.17)	14	(0.25)	1	(0.05)	0		2.05E-01	1.50E-01	1
	by sex:											
	• female	13	(0.14)	12	(0.22)	1	(0.05)	0		-	-	-
	• male	2	(0.02)	2	(0.04)	0		0		-	-	-

ACMG SF v2.0 (59 genes)										
All genes	438	(4.84)	272	(4.94)	91	(4.69)	75	(4.66)	1	1	1
AD genes	238	(2.63)	148	(2.69)	50	(2.58)	40	(2.49)	9.15E-01	9.15E-01	9.15E-01
by no. variants:											
• 1 P/LP	236	(2.61)	147	(2.67)	49	(2.52)	40	(2.49)	-	-	-
• 2 P/LP	2	(0.02)	1	(0.02)	1	(0.05)	0		-	-	-
by disease domain:											
• Cancer pred.	85	(0.94)	51	(0.93)	18	(0.93)	16	(1.00)	1	1	1
• CVD	73	(0.81)	34	(0.62)	26	(1.34)	13	(0.81)	**1.34E-02**	3.86E-01	2.21E-01
• Lipid disorder	65	(0.72)	58	(1.05)	3	(0.15)	4	(0.25)	**7.93E-05**	**1.70E-03**	7.09E-01
AR genes [a]	195	(2.15)	114	(2.07)	43	(2.22)	38	(2.36)	8.22E-01	8.22E-01	8.22E-01
XL genes	15	(0.17)	14	(0.25)	1	(0.05)	0		2.05E-01	1.50E-01	1
by sex:											
• female	13	(0.14)	12	(0.22)	1	(0.05)	0		-	-	-
• male	2	(0.02)	2	(0.04)	0		0		-	-	-

[a] No homozygous or compound heterozygous individuals with P/LP variants identified

Supplementary Table 4: Median values of genetic ancestral components of individuals with mismatched self-reported race/ethnicity (R/E) and ADMIXTURE-inferred genetic ancestry.

Interquartile range (IQR) refers to the Q1 to Q3 values of ancestral component indicated.

Self-reported R/E	Admixture-inferred ancestry	No. individuals	Median of genetic ancestral component (IQR)					
			Chinese		Indian		Malay	
Chinese	Malay	8	0.16	(0.06 - 0.27)	0.09	(0.08 - 0.17)	0.65	(0.58 - 0.80)
Indian	Chinese	35	0.50	(0.47 - 0.52)	0.43	(0.39 - 0.44)	0.08	(0.06 - 0.12)
	Malay	27	0.13	(0.10 - 0.17)	0.32	(0.21 - 0.37)	0.52	(0.48 - 0.61)
Malay	Chinese	126	0.55	(0.50 - 0.66)	0.06	(0.03 - 0.11)	0.35	(0.22 - 0.42)
	Indian	44	0.15	(0.10 - 0.25)	0.49	(0.45 - 0.59)	0.31	(0.20 - 0.38)
Others	Chinese	6	0.55	(0.52 - 0.75)	0.08	(0.04 - 0.14)	0.32	(0.13 - 0.36)
	Indian	11	0.10	(0.06 - 0.28)	0.87	(0.56 - 0.91)	0.04	(0.02 - 0.11)
	Malay	11	0.13	(0.05 - 0.18)	0.08	(0.06 - 0.15)	0.82	(0.15 - 0.68)

Supplementary Table 5: Association of the average fraction of ancestral component among individuals of Chinese, Indian and Malay ancestry groups with carriage status of pathogenic/likely pathogenic variants identified recurringly with the specified ancestral component.

Difference in ancestral component between variant carriers and non-carriers were evaluated by two-sided Wilcoxon rank-sum test. Adjusted p < 0.05 were considered significant. indv.: individuals

Ancestral component (A)	Genetic ancestry group	Carrier status of variant concordant with ancestral component (A)	Concordance of variant carrier status with genetic ancestry	No. indv.	Median ancestral component (A)	Difference in median ancestral component (Carrier vs Non-carrier)	Adjusted *p* value	Fold difference in median ancestral component (Carrier : Non-carrier)
Chinese	Chinese	Carrier	Concordant	455	0.895	0.012	**1.96E-03**	1.01
		Non-carrier	-	5047	0.882			
	Indian	Carrier	Discordant	2	0.321	0.258	**3.91E-02**	5.09
		Non-carrier	-	1939	0.063			
	Malay	Carrier	Discordant	26	0.275	0.141	**1.18E-07**	2.05
		Non-carrier	-	1582	0.134			
Indian	Chinese	Carrier	Discordant	3	0.008	-0.002	9.73E-01	0.76
		Non-carrier	-	5499	0.010			
	Indian	Carrier	Concordant	147	0.871	0.004	1.63E-01	1.01
		Non-carrier	-	1794	0.866			
	Malay	Carrier	Discordant	19	0.177	0.089	**1.42E-02**	2.01
		Non-carrier	-	1589	0.088			
Malay	Chinese	Carrier	Discordant	2	0.238	0.141	5.06E-02	2.44
		Non-carrier	-	5500	0.098			
	Indian	Carrier	Discordant	2	0.250	0.173	**4.87E-02**	3.28
		Non-carrier	-	1939	0.076			
	Malay	Carrier	Concordant	24	0.723	-0.028	9.73E-01	0.96
		Non-carrier	-	1584	0.751			

Supplementary Table 6: SG10K_Health individuals at-risk of Centers for Disease Control and Prevention (CDC) Tier 1 genetic conditions and their respective pharmacogenetic profile for the drugs commonly prescribed for their condition.

Individuals at-risk of CDC Tier 1 (CDC T1) genetic conditions were defined as those with P/LP variants in genes associated with the conditions (Hereditary breast and ovarian cancer (HBOC): *BRCA1, BRCA2, PALB2*; Lynch syndrome (LS): *MLH1, MSH2, MSH6, PMS2*; Familial hypercholesterolemia (FH): *APOB, LDLR, PCSK9*). CH: Chinese, IND: Indian, MY: Malay, N.A.: not available, NM: normal metabolizer, IM: intermediate metabolizer, PM: poor metabolizer, PGX: pharmacogenomic.

CDC T1 disorder	Drug	Pharmacogene with PharmGKB Level 1 evidence drug interaction	Pharmaco-phenotype	No. pharmacophenotype carriers				No. PGX risk allele carriers				Proportion of PGX risk allele carriers over disease at-risk individuals (%)			
				CH	IND	MY	Total	CH	IND	MY	Total	CH	IND	MY	Total
HBOC	Tamoxifen	*CYP2D6*	NM	15	9	6	30								
			IM	9	1	2	12	10	1	3	14	28.6	8.3	21.4	23.0
			PM	1	0	1	2								
			N.A. [a]	10	2	5	17								
			Total (at-risk)	35	12	14	61								
LS	Irinotecan	*UGT1A1*	NM	7	0	1	8								
			IM	4	2	1	7	5	2	1	8	38.5	100.0	50.0	47.1
			PM	1	0	0	1								
			N.A. [a]	1	0	0	1								
			Total (at-risk)	13	2	2	17								
FH	Statins drugs	*SLCO1B1*	NM	46	3	4	53								
			IM	10	0	0	10	10	0	0	10	17.2	0	0	15.4
			PM	0	0	0	0								
			N.A. [a]	2	0	0	2								
			Total (at-risk)	58	3	4	65								
Overall							143				32				22.4

[a] Pharmacophenotype not available due to unavailable genotype.

SUPPLEMENTARY NOTE

Study Cohort Acknowledgements

We acknowledge the contributing investigators from the GUSTO study group: Allan Sheppard, Amutha Chinnadurai, Anne Eng Neo Goh, Anne Rifkin-Graboi, Anqi Qiu, Arijit Biswas, Bee Wah Lee, Birit F.P. Broekman, Boon Long Quah, Borys Shuter, Chai Kiat Chng, Cheryl Ngo, Choon Looi Bong, Christiani Jeyakumar Henry, Claudia Chi, Cornelia Yin Ing Chee, Yam Thiam Daniel Goh, Doris Fok, E Shyong Tai, Elaine Tham, Elaine Quah Phaik Ling, Evelyn Chung Ning Law, Evelyn Xiu Ling Loo, Fabian Yap, Falk Mueller-Riemenschneider, George Seow Heong Yeo, Helen Chen, Heng Hao Tan, Hugo P S van Bever, Iliana Magiati, Inez Bik Yun Wong, Ivy Yee-Man Lau, Izzuddin Bin Mohd Aris, Jeevesh Kapur, Jenny L. Richmond, Jerry Kok Yen Chan, Joanna D. Holbrook, Joanne Yoong, Joao N. Ferreira, Jonathan Tze Liang Choo, Jonathan Y. Bernard, Joshua J. Gooley, Keith M. Godfrey, Kenneth Kwek, Kok Hian Tan, Krishnamoorthy Niduvaje, Kuan Jin Lee, Leher Singh, Lieng Hsi Ling, Lin Lin Su, Ling-Wei Chen, Lourdes Mary Daniel, Lynette P Shek, Marielle V. Fortier, Mark Hanson, Mary Foong-Fong Chong, Mary Rauff, Mei Chien Chua, Melvin Khee-Shing Leow, Michael Meaney, Mya Thway Tint, Neerja Karnani, Ngee Lek, Oon Hoe Teoh, P. C. Wong, Paulin Tay Straughan, Peter D. Gluckman, Pratibha Agarwal, Queenie Ling Jun Li, Rob M. van Dam, Salome A. Rebello, Seang-Mei Saw, See Ling Loy, S. Sendhil Velan, Seng Bin Ang, Shang Chee Chong, Sharon Ng, Shiao-Yng Chan, Shirong Cai, Shu-E Soh, Sok Bee Lim, Stella Tsotsi, Chin-Ying Stephen Hsu, Sue Anne Toh, Swee Chye Quek, Victor Samuel Rajadurai, Walter Stunkel, Wayne Cutfield, Wee Meng Han, Wei Wei Pang, Yap-Seng Chong, Yin Bun Cheung, Yiong Huak Chan and Yung Seng Lee.

SUPPLEMENTARY REFERENCES

1. Soh, S.-E. *et al.* Cohort profile: Growing Up in Singapore Towards healthy Outcomes (GUSTO) birth cohort study. *Int. J. Epidemiol.* **43**, 1401–1409 (2014).
2. Tan, K. H. X. *et al.* Cohort Profile: The Singapore Multi-Ethnic Cohort (MEC) study. *Int. J. Epidemiol.* **47**, 699–699j (2018).
3. Bylstra, Y. *et al.* Implementation of genomics in medical practice to deliver precision medicine for an Asian population. *Npj Genomic Med.* **4**, 1–7 (2019).
4. Majithia, S. *et al.* Cohort Profile: The Singapore Epidemiology of Eye Diseases study (SEED). *Int. J. Epidemiol.* **50**, 41–52 (2021).
