# Supplementary material for: Analysis of clinically relevant variants from ancestrally diverse Asian genomes
Source: Nat Commun. 2022 Nov 5;13:6694. doi: 10.1038/s41467-022-34116-9 (PMC9637116; doi:10.1038/s41467-022-34116-9)
Supplement: Supplementary file 3 — Description of Additional Supplementary Files [file 41467_2022_34116_MOESM3_ESM.pdf]

Description of Additional Supplementary Files

Filename: Supplementary Data 1
Description:
Gene-level carrier frequencies for genes with P/LP variants identified. For genes with total carriers ≥5, differences in carrier frequencies across ancestries were tested using two-sided Fisher's exact test with Benjamini-Hochberg correction for multiple testing. Sample size of the three SG10K_Health ancestry groups are Chinese (CH): 5502, Indian (IND): 1941, Malay (MY): 1608. AD: autosomal dominant, AR: autosomal recessive, DD: digenic dominant, DR: digenic recessive, XL: X-linked, XLR: X-linked recessive, Y: gene is included in the specific gene panel/recommendation list, N: gene is not included in the specific gene panel/recommendation list. [#] The highest carrier frequency among the three SG10K_Health ancestry groups.

Filename: Supplementary Data 2
Description:
Curated pathogenic/likely pathogenic (P/LP) variants identified from SG10K_Health analyzed cohort (unrelated to the second degree). Sample size of the three SG10K_Health ancestry groups are Chinese (CH): 5502, Indian (IND): 1941, Malay (MY): 1608. AC: allele count, AF: allele frequency, AN: allele number, AFR: African, AMR: admixed American, ASJ: Ashkenazi Jewish, EAS: East Asian, NFE: Non-Finnish European, SAS: South Asian, nHet: count of allele in heterozygous state, nHom: count of allele in homozygous state. [a] Variant is specific to the indicated ancestry group based on local ancestry inference, as described in Methods, and is used for analysis in Figure 2B.

Filename: Supplementary Data 3
Description:
Carrier frequencies for variants in autosomal dominant disorder genes in ACMG SF v3.0 for the subset of SG10K_Health cohort aged 50 years and younger. Sample size of the three SG10K_Health ancestry groups are Chinese (CH): 2977, Indian (IND): 1332, Malay (MY): 1173. Statistical analysis was performed for variants with carrier frequency of at least 0.1%. Highlighted bold fonts are statistically significant p values ($p < 0.05$) for two-sided Fisher's exact test. Adjusted p values were derived using Benjamini-Hochberg correction.

Filename: Supplementary Data 4
Description:
Carrier frequency of copy number deletions identified in loss-of-function intolerant (LOFi) genes by ancestry group in SG10K_Health.

Filename: Supplementary Data 5
Description:
List of 106 variants of uncertain significance-favour pathogenic (VUS-FP) identified in genes of ACMG SF v3.0 list. *In silico* criteria for predicting pathogenic potential is detailed in Methods. AC: allele count, AF: allele frequency, AN: allele number, AFR: African, AMR: admixed American, ASJ: Ashkenazi Jewish, EAS: East Asian, NFE: non-Finnish European, SAS: South Asian.

[a] number of variants with pathogenic/likely pathogenic classification in ClinVar with at least two star rating within 25bp rolling window
[b] number of variants with benign/likely benign classification in ClinVar with at least two star rating within 25bp rolling window
[c] number of protein-truncating variants (PTV, including nonsense, frameshift, canonical splice variants) in ClinVar with at least two star ratings for the gene
[d] total number of heterozygous and hemizygous carriers

Filename: Supplementary Data 6
Description:
Allele frequencies of risk alleles identified in the SG10K_Health cohort for the 23 pharmacogenes analyzed.

Filename: Supplementary Data 7
Description:
The overall and ancestry-specific frequency of carriers for pharmacophenotypes with a therapeutic recommendation (per CPIC guidelines). [a] As recommended by CPIC guidelines/PharmGKB.

Filename: Supplementary Data 8
Description:
Novel putative loss-of-function variants pharmacogenetic variants. AF: allele frequency, AC: allele count, AN: allele number, nHet: number of heterozygous carriers, nHom: number of homozygous carriers, nHemi: number of hemizygous carriers.

Filename: Supplementary Data 9
Description:
List of 4143 genes associated with autosomal dominant, autosomal recessive and X-linked monogenic disorders consolidated from Genomics England PanelApp diagnostic-grade (green) status panel, OMIM and in-house gene panels. Genes identified as loss-of-function intolerant (LOFi) by any one of the three critieria (described in Methods) are indicated.

Filename: Supplementary Data 10
Description:
List of CPIC gene-drug pairs with PharmGKB Level 1A/1B for the 23 pharmacogenes included in analysis.

Filename: Supplementary Data 11
Description:
Carrier frequency of diplotypes associated with actionable pharmacogenomic outcomes for the pharmacogenes with star allele nomenclature. CH: Chinese, IND: Indian, MY: Malay
