# Supplementary material for: Analysis of clinically relevant variants from ancestrally diverse Asian genomes
Source: Nat Commun. 2022 Nov 5;13:6694. doi: 10.1038/s41467-022-34116-9 (PMC9637116; doi:10.1038/s41467-022-34116-9)
Supplement: Supplementary file 5 — Reporting Summary [file 41467_2022_34116_MOESM5_ESM.pdf]

nature portfolio

Corresponding author(s): Weng Khong Lim

Last updated by author(s): Sep 10, 2022

Reporting Summary

Nature Portfolio wishes to improve the reproducibility of the work that we publish. This form provides structure for consistency and transparency in reporting. For further information on Nature Portfolio policies, see our Editorial Policies and the Editorial Policy Checklist.

Statistics

For all statistical analyses, confirm that the following items are present in the figure legend, table legend, main text, or Methods section.

n/a	Confirmed	
☐	☒	The exact sample size (*n*) for each experimental group/condition, given as a discrete number and unit of measurement
☐	☒	A statement on whether measurements were taken from distinct samples or whether the same sample was measured repeatedly
☐	☒	The statistical test(s) used AND whether they are one- or two-sided<br>*Only common tests should be described solely by name; describe more complex techniques in the Methods section.*
☐	☒	A description of all covariates tested
☐	☒	A description of any assumptions or corrections, such as tests of normality and adjustment for multiple comparisons
☐	☒	A full description of the statistical parameters including central tendency (e.g. means) or other basic estimates (e.g. regression coefficient) AND variation (e.g. standard deviation) or associated estimates of uncertainty (e.g. confidence intervals)
☐	☒	For null hypothesis testing, the test statistic (e.g. *F*, *t*, *r*) with confidence intervals, effect sizes, degrees of freedom and *P* value noted<br>*Give P values as exact values whenever suitable.*
☒	☐	For Bayesian analysis, information on the choice of priors and Markov chain Monte Carlo settings
☒	☐	For hierarchical and complex designs, identification of the appropriate level for tests and full reporting of outcomes
☐	☒	Estimates of effect sizes (e.g. Cohen's *d*, Pearson's *r*), indicating how they were calculated

Our web collection on statistics for biologists contains articles on many of the points above.

Software and code

Policy information about availability of computer code

Data collection	No software was used for data collection, as this was an opportunistic study.
Data analysis	Sequencing data were jointly-processed in a standardized bioinformatics pipeline that made use of the following tools detailed in Methods: Burrows-Wheeler Aligner (BWA-MEM, v0.7.17), GenomeAnalysisToolKit (GATK, v4.0.6.0). Variants were annotated using Ensembl Variant Effect Predictor (VEP, release 100.0). Variant curation made use of freely available tools including AutoPVS1 (v1.1), CardioClassifier (v.0.2.0), Integrative Genomics Viewer (IGV, v2.8.2). Structural variants were analysed using Manta (v1.6), svimmer (v0.1), graphtyper (v2.5.1), duphold (v0.2.3), samplot (v1.0.20), SMNCopyNumberCaller (v1.1.1). Kinship analysis, global and local ancestry inference made used of tools including Somalier (v0.2.13), PLINK (v1.90b3.46), Kinship-based Inference for Genome-wide association studies (KING, ver 2.2.3), ADMIXTURE (ver 1.3.0), EAGLE (v2.4.1), RFMix (v2.03-r0) as detailed in Methods. Pharmacogenetic variants were identified using Cyrius (v1.0), Aldy (v3.1), HLA-HD (v1.3.0) as defined in Methods. Statistical analyses were performed using R (v4.1.0).<br><br>All code to perform all analyses and regenerate all the figures in this manuscript is provided at https://github.com/csockhoai/SG10KMed and released at https://doi.org/10.5281/zenodo.7057754.

For manuscripts utilizing custom algorithms or software that are central to the research but not yet described in published literature, software must be made available to editors and reviewers. We strongly encourage code deposition in a community repository (e.g. GitHub). See the Nature Portfolio guidelines for submitting code & software for further information.

Data

Policy information about availability of data

All manuscripts must include a data availability statement. This statement should provide the following information, where applicable:

- Accession codes, unique identifiers, or web links for publicly available datasets
- A description of any restrictions on data availability
- For clinical datasets or third party data, please ensure that the statement adheres to our policy

Source data for all analyses and regeneration of all display items contain individual-level data including genotypes, which is made available to researchers registered through the SG10K_Health Data Access Portal (https://www.npm.sg/collaborate/partners/sg10k/). Data for this study were obtained under Data Access Application NPM00003. Data from the publicly available gnomAD (v3.1), ClinVar (accessed September 9 2020), CPIC (accessed Aug 30 2021), PharmVar (accessed April 2021) were referenced for variant analyses.

Field-specific reporting

Please select the one below that is the best fit for your research. If you are not sure, read the appropriate sections before making your selection.

☒ Life sciences ☐ Behavioural & social sciences ☐ Ecological, evolutionary & environmental sciences

For a reference copy of the document with all sections, see nature.com/documents/nr-reporting-summary-flat.pdf

Life sciences study design

All studies must disclose on these points even when the disclosure is negative.

Sample size	Sample size was not predetermined, as this study was opportunistic and involved secondary use of generated whole genome data.
Data exclusions	Dataset analysed excluded individuals related to the second degree inferred through kinship analysis.
Replication	We attempted replication of findings from a subset of our population (Chinese, Indian) closest to the gnomAD database populations (EAS, SAS) as reported in Results. Replication was performed once and was successful. There is no comparable dataset for our Malay population for replication.
Randomization	This is not relevant to our study because this is not a case-control study, hence randomization was not performed.
Blinding	This is not relevant to our study because this is not a case-control study, hence blinding was not performed.

Reporting for specific materials, systems and methods

We require information from authors about some types of materials, experimental systems and methods used in many studies. Here, indicate whether each material, system or method listed is relevant to your study. If you are not sure if a list item applies to your research, read the appropriate section before selecting a response.

Materials & experimental systems

n/a	Involved in the study	
☒	☐	Antibodies
☒	☐	Eukaryotic cell lines
☒	☐	Palaeontology and archaeology
☒	☐	Animals and other organisms
☐	☒	Human research participants
☒	☐	Clinical data
☒	☐	Dual use research of concern

Methods

n/a	Involved in the study	
☒	☐	ChIP-seq
☒	☐	Flow cytometry
☒	☐	MRI-based neuroimaging

Human research participants

Policy information about studies involving human research participants

Population characteristics	As this was an opportunistic study on a cross-sectional population, participants included in the analysis were not selected for any specific characteristics. The covariate-relevant characteristics used in the study included age, sex, self-reported race/ethnicity, genetic ancestry, LDL cholesterol levels, lipid-lowering medication intake.
Recruitment	We did not recruit any participants for this study. The dataset analysed in this study is derived from SG10K_Health, aggregated from six participating studies. Individuals in all studies were recruited with signed informed consent from the

	participating individual or parent/guardian in the case of minors, according to the respective study protocols listed in Supplementary Table 1.
Ethics oversight	The dataset analysed in this study is a secondary use of an aggregated dataset of six participating studies. Participants in all studies were recruited with signed informed consent according to the respective study protocols and all studies were approved by relevant institutional ethics review board detailed in Supplementary Table 1.

Note that full information on the approval of the study protocol must also be provided in the manuscript.
